# A Novel *Meloidogyne incognita* Effector Misp12 Suppresses Plant Defense Response at Latter Stages of Nematode Parasitism

**DOI:** 10.3389/fpls.2016.00964

**Published:** 2016-06-30

**Authors:** Jialian Xie, Shaojun Li, Chenmi Mo, Gaofeng Wang, Xueqiong Xiao, Yannong Xiao

**Affiliations:** Key Laboratory of Plant Pathology of Hubei Province, College of Plant Science & Technology, Huazhong Agricultural UniversityWuhan, China

**Keywords:** *Misp12*, *Meloidogyne incognita*, effector protein, defense suppression, latter parasit

## Abstract

Secreted effectors in plant root-knot nematodes (RKNs, or *Meloidogyne* spp.) play key roles in their parasite processes. Currently identified effectors mainly focus on the early stage of the nematode parasitism. There are only a few reports describing effectors that function in the latter stage. In this study, we identified a potential RKN effector gene, *Misp12*, that functioned during the latter stage of parasitism. *Misp12* was unique in the *Meloidogyne* spp., and highly conserved in *Meloidogyne incognita*. It encoded a secretory protein that specifically expressed in the dorsal esophageal gland, and highly up-regulated during the female stages. Transient expression of *Misp12-GUS-GFP* in onion epidermal cell showed that Misp12 was localized in cytoplast. In addition, *in planta* RNA interference targeting *Misp12* suppressed the expression of *Misp12* in nematodes and attenuated parasitic ability of *M. incognita.* Furthermore, up-regulation of jasmonic acid (JA) and salicylic acid (SA) pathway defense-related genes in the virus-induced silencing of *Misp12* plants, and down-regulation of SA pathway defense-related genes in *Misp12*-expressing plants indicated the gene might be associated with the suppression of the plant defense response. These results demonstrated that the novel nematode effector *Misp12* played a critical role at latter parasitism of *M. incognita*.

## Introduction

Root-knot nematodes (RKNs, or *Meloidogyne* spp.) are a large kind of plant-parasite nematodes. It is thought that they could infest more than 5,000 plant species (Blok et al., 2008) and are responsible for substantial economic crop loss (Elling, 2013). As a major specie of RKNs, *Meloidogyne incognita* has a remarkably wide host range, including some principal crops, and causes more economical loss than other species (Trudgill and Blok, 2001). Elucidating the mechanisms of RKNs parasitism would facilitate the development of new control strategies for nematode diseases.

As sedentary parasites, RKNs usually establish a feeding site composed of giant cells (GCs) to obtain nutrients from plants (Bird et al., 2009). During the process of nematodes infection, proteins secreted through the stylet of RKNs induce vascular cells nuclear division without cytokinesis and ultimately transform four to eight root cells into the GCs (Jaubert et al., 2004; Huang et al., 2006; Caillaud et al., 2008). Although the molecular pathogenic mechanisms of RKNs are still largely uncharacterized, it is believed that proteins synthesized in oesophageal glands and injected through the stylet into plant tissue play key roles in the parasitism of nematodes (Hewezi and Baum, 2013). These secretory proteins function as effectors in GCs formation and maintenance to support the nematode parasitism (Davis et al., 2000; Quentin et al., 2013). Some cell wall-degrading and cell wall-modifying enzymes, such as β-1,4-endoglucanase, β-1,4-endoxylanase, pectate lyase, and cellulose-binding protein, have been characterized as potential effectors involved in the invasion of root tissues by preparasitic juveniles and the migration of nematodes (Ding et al., 1998; Rosso et al., 1999; Popeijus et al., 2000; Dautova et al., 2001; Doyle and Lambert, 2002; Huang et al., 2005a; Ledger et al., 2006). Some effectors including chorismate mutase, venom allergen-like protein, and glutathione S-transferase, are involved in the suppression of defense reactions of the host cell during the infection stages (Lambert et al., 1999; Ding et al., 2000; Huang et al., 2005b; Long et al., 2006; Dubreuil et al., 2007; Wang et al., 2007). Some effectors, such as *MiMsp40* and *MeTCTP*, can suppress programmed cell death (PCD) in host plants to promotes parasitism (Niu et al., 2016; Zhuo et al., 2016). Notably, recently identified effectors function in disturbing the cells metabolism. The *M. incognita* effector *Mi8D05* was proved to play an important role in the regulation of solute and water transport within GCs (Xue et al., 2013), and *M. javanica* effector *MjTTL5* could activate the host reactive oxygen species-scavenging system(Lin et al., 2016). Additionally, some effectors, including *MiEFF1, MiCRT, MjNULG1*, and *7H08*, were found to target the host plant cell nuclei, manipulate the host cell processes and exhibit the transcriptional activation activity (Jaouannet et al., 2012, 2013; Lin et al., 2013; Zhang et al., 2015a).

To identify more novel effectors, transcriptomic approaches were used to analyze the secreted genes from the microaspiration of esophageal gland cells (Gao et al., 2003; Huang et al., 2003). Since the genome of *M. incognita* and *M. hapla* were sequenced (Abad et al., 2008; Opperman et al., 2008), the increasing genomic data has provided a convenient way to identify RKNs effector proteins and other essential genes.

The studies focusing on the effectors not only promotes an understanding of nematode-host interaction mechanisms, but also screens new target genes that could be applied for nematocides and breeding of nematode resistant plants to control nematode diseases. The RNA interference of the effector gene *Mc16D10L* confers resistance against *M. chitwood* in *Arabidopsis* and potato plants (Dinh et al., 2014), and the suppression of *NGB* and *NAB/ERabp1* in tomatoes resulted in the reduction in the number of *Globodera rostochiensis* (Dąbrowska-Bronk et al., 2014).

In this study, combined with the genomic sequences, proteins, an EST library of *M. incognita* and bioinformatics tools, a potential effector *Misp12* (*M. incognita* putative esophageal gland cell secretory protein 12) was selected. After BLAST against NCBI database, we found that this gene was also pointed out via transcriptomic approaches and was named *Msp12* (Huang et al., 2003). We further analyzed the developmental expression profiles and investigated subcellular location *in planta* of *Misp12.* In addition, the VIGS (virus induced gene silencing) approaches and the transiently expression of *Misp12* in plants were also carried out to examine the function of *Misp12* during *M. incognita* parasitism.

## Materials and Methods

### Nematodes and Plants

*Meloidogyne incognita* were collected and identified from 8 different areas of P. R. China and reared on tomato plants in greenhouses at 25°C. Pre-parasitic second-juveniles (J2s) and parasitic stages were collected as described previously (Huang et al., 2005a). *Lycopersicon esculentum* and *Nicotiana benthamiana* plants routinely grow in pots at 25°C in the greenhouse.

### Nucleic Acid Extraction and RT-PCR Analysis

Genomic DNA was extracted from *M. incognita* eggs and pre-parasitic J2s by using the cetyltrimethylammonium bromide (CTAB) method, as described by Goetz et al. (2001). Total RNA of nematodes was isolated using the MiniBEST Universal RNA Extraction Kit (TaKaRa, Dalian, China) after grinding nematodes in a 1.5 mL sterile tube with liquid nitrogen and then treated by *DNase* I (Thermo Scientific, Shanghai, China) at 37°C for 30 min to remove genomic DNA. First-strand cDNA was synthesized using the SuperScript^®^ III Reverse Transcriptase kit (Invitrogen, Shanghai, China). Quantitative real-time reverse transcription-PCR (qRT-PCR) was performed on CFX96^TM^ Real TimeSystem (BIO-RAD, USA) with the following conditions: 95°C for 30 s and 40 cycles of 95°C for 10 s, 60°C for 20 s, and 72°C for 30 s, using the SYBR Green PCR Master Mix (TaKaRa, Dalian, China). Quantification of the relative changes in gene expression was performed using the 2^-ΔΔCT^ method (Livak and Schmittgen, 2001). The experiments were repeated three times, with three technical replicates for each reaction.

### Sequence Analysis and Gene Cloning

The whole genome and protein sequences of *M. incognita* were downloaded from the *M. incognita* resources database ^1^. The EST library of *M. incognita* was obtained from NCBI. SignalP 3.0 (Bendtsen et al., 2004), TMHMM 2.0 (Krogh et al., 2001), and MERCI (Vens et al., 2011) were used to predict potential effector proteins.

The 3′-RACE-Ready cDNA was synthesized from 1 μg of total RNA using the BD SMART RACE cDNA amplification kit (TaKaRa, Dalian, China). Based on the predicted sequence of *Misp12* described above, the primers RACE12S1 and RACE12S2 were designed. The 3′-terminal sequence was amplified by PCR using RACE12S1 and the 3′-anchor UPM primes, followed by a second-round PCR with RACE12S2 and NUP primers using the first-round PCR products as template. PCR was performed following the BD SMART RACE cDNA amplification kit user manual.

To confirm the predicted sequence, primers covering the whole sequence, Misp12QS and Misp12QA were designed to perform PCR from cDNA and DNA templates. For PCR amplification, 0.1 μg of cDNA or DNA template was used in a 50 μl reaction mixture consisting of 1 × PCR buffer for Phusion High-Fidelity DNA Polymerase, 0.2 mM of each dNTP, 1.5 mM MgSO_4_, 0.3 μM primers, and 2 units of Phusion High-Fidelity DNA Polymerasee (Thermo Scientific, Shanghai, China). PCR conditions were as follows: predenaturation at 94°C for 3 min; 35 cycles of denaturation at 94°C for 45 s, annealing at 57°C for 45 s, and polymerization at 72°C for 90 s; with a final incubation step at 16°C. All primers used in this study were synthesized by Invitrogen Biotechnology Co. Ltd. (Shanghai, China) and are listed in Supplementary Table S1.

### Sequence Comparisons and Secondary Structure Predictions

The signal peptide and its cleavage site were predicted by the SignalP 3.0 (Bendtsen et al., 2004) and the TargetP 1.0 (Emanuelsson et al., 2000). Calculation of the predicted *Misp12* molecular weight and isoelectric point were performed using the ProtParam program (Gasteiger et al., 2005). Secondary structure prediction of the protein sequence was performed using the PHD program (Rost and Sander, 1993) and the motifs were predicted by Motif Scan (Pagni et al., 2007). The ClustalW program (Thompson et al., 1994) was used to generate an alignment of *M. incognita* nematodes in eight different areas of China. The HHPred program (Remmert et al., 2011) was used to predict conserved domains.

### Developmental Expression Analyses

Total RNA samples were prepared from 200 *M. incognita* nematodes at different life stages by the method described above. The expression level of *Misp12* was analyzed via qRT-PCR with the primes qPCRmi12S/qPCRmi12A. Total cDNA abundance in the samples was normalized using the *Actin* as controls, which were amplified by primes QactinS/QactinA. These experiments were repeated three times, with three technical replicates for each reaction.

### *In Situ* mRNA Hybridization

*In situ* hybridization was performed as previously described with a slight modification (de Boer et al., 1998). The hybridization temperature was adjusted to 48°C. Primers Misp12HS and Misp12HA (Supplementary Table S1) were employed to synthesize digoxigenin (DIG)-labeled sense and antisense cDNA probes (Roche, USA) by asymmetric PCR. The sense cDNA probes serve as control (Huang et al., 2003).

### Subcellular Localization of Misp12

The open reading frame (ORF) of *Misp12*, with and without a signal peptide sequence, was cloned with primes 12S/12BamHI-A and 12nospS/12nospBamHI-A, respectively (Supplementary Table S1). The *GUS* gene was cloned with primer pairs GusBamHI-S/GusA. After *BamHI* digestion, the *Misp12* and the *GUS* gene were ligated together. Then, the Misp12:GUS and ^Δsp^Misp12:GUS were cloned with primer pairs12EcoRIS/GusHindIIIA. The sequenced PCR products were digested with *EcoR*I and *Hind*III and then ligated into *EcoR*I/*Hind*III-digested vector pEGAD (Cutler et al., 2000) to generate the vectors 35S:Misp12:GUS:eGFP and 35S:^Δsp^Misp12:GUS:eGFP. The recombinant vectors were transferred into the *Agrobacterium tumefaciens* GV3101 using standard cloning techniques (Sambrook and MacCallum, 2012). Healthy and fresh onion scales (1–1.5 cm × 1 cm) were placed on a 9 cm plate and their inner surfaces were immersed into 6 mL resuspension of *A. tumefaciens* solution (OD600 = 1–1.5) consisting of 5% (g/v) sucrose, 100 mg L^-1^ acetosyringone and 0.02% (v/v) Silwet-77 for 6–12 h at 28°C. Then, the onion scales were transferred to a petri dish containing 25 mL 1/2 MS (Murashige and Skoog salts, 30 g L^-1^ sucrose and 0.7% (g/v) agar, pH 5.7) for 1–2 days. The subcellular localization of the fused proteins was visualized using fluorescence microscopy (Nikon 80i, Nikon, Japan) at an excitation wavelength of 488 nm.

### *In Planta* RNAi

The tobacco rattle virus (TRV)-based vectors pTRV_1_ and pTRV_2_ were used for gene expression in *N. benthamiana* as previously described (Liu et al., 2002). The unique 249 bp fragment of the gene *Misp12* was amplified with the primer pairs U12RNAiS/U12RNAiA. The PCR product was digested with *Xba*I and *Sac*I and then inserted into pTRV_2_ to generate vector pTRV_2_::*Misp12*. The vectors pTRV_1_, pTRV_2_::00 (negative control), and pTRV_2_::*Misp12* were transferred into the *Agrobacterium tumefaciens* GV3101, respectively. *A. tumefaciens* cultures containing pTRV_1_ and pTRV_2_::*Misp12* were injected into *Nicotiana benthamiana* as the previously described (Xiao et al., 2014), while *A. tumefaciens* cultures containing pTRV_1_ and pTRV_2_::00 were used as controls. Five days after inoculation (DAI), primer pairs TRVcpF/TRVcpR (Anand et al., 2007) were used to test the TRV coat protein transcription by qRT-PCR to check whether TRV invasion was successful.

To detect the parasitism ability of *Misp12*-RNAi nematodes, 150 *N. benthamiana* plants agroinfiltrated with pTRV::*Misp12* or pTRV::00 were inoculated with 300 pre-J2 nematodes, respectively. Additionally, 150 untreated plants were used as blank controls. At 5, 10, 15, and 28 DAI, tomato plant roots were stained with byacid fuchsin to count the numbers of parasitic nematodes (Bybd et al., 1983). At 45 DAI, the galls and eggs in tomato plant roots were counted. Ten *N. benthamiana* plants were checked at each time point. The same experiments were repeated three times. Statistically significant differences between each treatments and the corresponding control were determined by Student’s *t*-test using SAS version 9.0.

To evaluate the *Misp12* expression level in gene-RNAi nematodes, mRNA was extracted from mix-stages nematodes isolated from *Misp12*-RNAi and control plants at 15 DAI and 28 DAI. Each treatment was sampled three times. The relative expression level of *Misp12* was checked with the primer pair qPCRmi12S/qPCRmi12A by qRT-PCR as described above.

### Transiently Expressing Misp12 in Plants

The suppression of PCD in *N. benthamiana* leaves was assessed as previously described (Bos et al., 2006). The ORF of *Misp12*, with and without a signal peptide sequence, was cloned into the PVX vector pGR107, respectively (Article et al., 2011). The *INF1* gene was amplified from *Phytophthora infestans* isolate 88069 genomic DNA and cloned into pGR107 (Article et al., 2011).

The confirmed constructs were introduced into the *A. tumefaciens* strain GV3101 by electroporation. The cultured *A. tumefaciens* cells (OD_600_ = 0.4) carrying *Misp12* and ^Δsp^*Misp12* were initially infiltrated into the leaves of *N. benthamiana* plants, which were grown in the greenhouse for 6 weeks at 25–28°C under 16 h light/8 h dark. The identical infiltration site was then challenged with *A. tumefaciens* cells carrying the *INF*1 gene at 24 h after initial inoculation. Simultaneously, the *INF1* gene was expressed alone as controls. The plants were monitored for symptoms, images were acquired 2 days after the last infiltration. The experiment was repeated at least three times, and each assay consisted of at least five plants with three leaves inoculated similarly.

### Detection of JA and SA Signaling Pathways in Plants

The jasmonic acid (JA) and salicylic acid (SA) signaling pathways genes expression levels were tested in the VIGS of *Misp12* plants at 28 DAI and *Misp12*-expressing plants at 2 DAI compared to their control plants by qRT-RCR, respectively.

The SA signaling pathways molecular markers were the transcript of pathogenesis-related gene *PR-1*(Accession no. JN247448) and phenylalanine ammonia lyase gene *PAL5* (Accession no. EB684217.1). For the activation of JA signaling pathways, transcripts of proteinase inhibitor gene *Pin2* (Accession no. EH368183.1), 12-oxophytodienoate reductase gene *OPR3* (Accession no. CN745683) and β*-thionin* gene (Accession no. EH368982.1) were analyzed. For all qRT-PCR analysis, transcripts were normalized against the geometric mean of the expression levels of two *N. benthamiana* reference genes *Actin* and *GAPDH* that were amplified with the two pairs of primes NbactinS/A and NbGAPDHS/A, respectively.

## Results

### Identification of the *Misp12*

The whole protein sequences of *M. incognita* were used the bioinformatics tools SignalP 3.0 and TMHMM 2.0 to predict the secreted proteins. Then, the MERCI tools were used to predict the potential effectors. *Misp12* was found to be a candidate secretory protein. After cloning the cDNA and the genome sequence of *Misp12* via 3′RACE and genome walking, it revealed that *Misp12* was 1202-bp in length, encompassing a 450 bp ORF (GeneBank: KU737535), which encoded a deduced protein of 149 amino acids.

**FIGURE 1:**
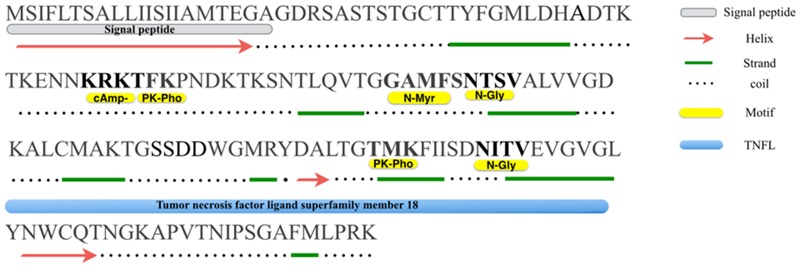
**Primary and secondary structures analysis of Misp12.** A putative hydrophobic leader/signal peptide, as predicted by the SignalP program is shown at the N terminus along with a cleavage site between two alanine residues at position 20–21. Consensus N-linked glycosylation site (*N*-gly), protein kinase C phophorylation sites (PK-pho), cAMP-and cGMP-dependent glycosylation site (cAmp-) and *N*-myristoylation site (*N*-Myr) are indicated.

SignalP and TargetP analysis results revealed that a signal peptide sequence existed at the N-terminal region, where a predicted cleavage site was located between amino acid positions A^19^ and A^20^ (**Figure 1**), generating a small mature protein with a theoretical molecular weight of 15.9 kDa and an isoelectric point of 8.81. Furthermore, no transmembrane region was detected by TMHMM2.0 (Krogh et al., 2001).

Based on PHD program analysis, we found that Misp12 consisted of 8 beta-strands and 2 helixes (except the signal peptide) (**Figure 1**). Motif Scan analysis revealed that two N-linked glycosylation sites presented at residues N^74^ and N^115^, two Protein kinase C phosphorylation sites at residues T^52^ and T^107^, a cAMP-and cGMP-dependent glycosylation site at residue K^49^, and an N-myristoylation site at residue G^69^ (**Figure 1**). HHPred analysis showed that Misp12 has a region similar to tumor necrosis factor ligand superfamily in the C-terminal between residues K^84^ and G^121^ (**Figure 1**).

In addition, no ortholog of *Misp12* was found in other organisms when searching in the NCBI database using BLAST. The ClustalW (Thompson et al., 1994) was used to align 9 *Misp12* alignments of *M. incognita* from eight different areas of China and 1 *Misp12* homolog sequence (accession no. AY134431.1) in NCBI, and the result showed that they have 99% identity, with 16 points mutations exist in the gene sequence and 12 of the 16 were in ORF, and only 2 are missense mutations (**Supplementary Figure S1**).

### The *Misp12* Gene Was Highly Expressed during the Nematode Mature Stage

**FIGURE 2:**
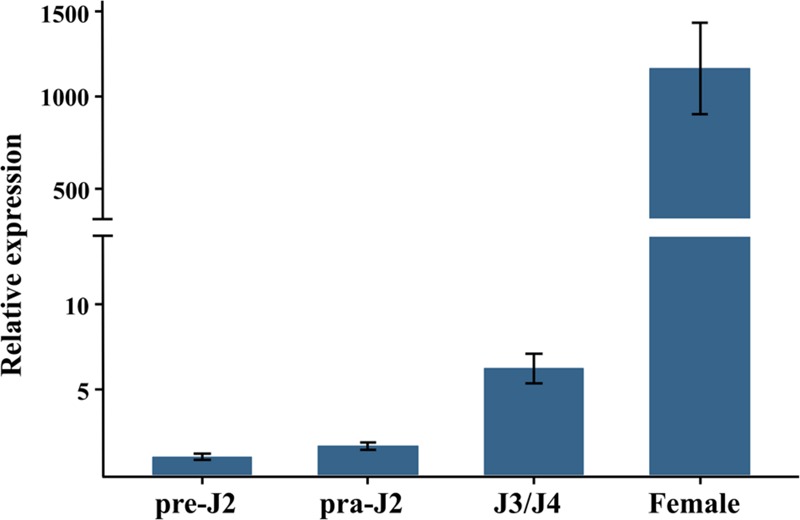
**Developmental expression pattern of *Misp12*.** The relative expression of *Misp12* was quantified using quantitative RT-PCR in four *M. incognita* life stages, pre-parasitic second-stage juvenile (pre-J2), parasitic second-juveniles (par-J2), third- and fourth-stage juveniles (J3/J4), and mature females. Fold change values were calculated using the 2^-ΔΔCT^ method and normalized to the *Meloidogyne incognita Actin* gene and relative to expression in pre-J2s. Each column represents the mean of three independent experiments with standard deviation.

To figure out which developmental stage *Misp12* mainly takes part in, the expression pattern of the gene was evaluated via qRT-PCR between non-parasitic stages and parasitic stages. The result showed that *Misp12* transcript accumulated the most in the mature female stage, which increased 1200-fold when compared to the non-parasitic stages (eggs and pre-parasitic J2s) (**Figure 2**). In addition, the expression level of *Misp12* at parasitic J2 stage and third- and fourth-stage juveniles (J3/J4) stage were also higher than non-parasitic stages (**Figure 2**). These results suggested that Misp12 was more important at latter parasitism.

### *Misp12* Was Specifically Expressed in the Dorsal Esophageal Gland

**FIGURE 3:**
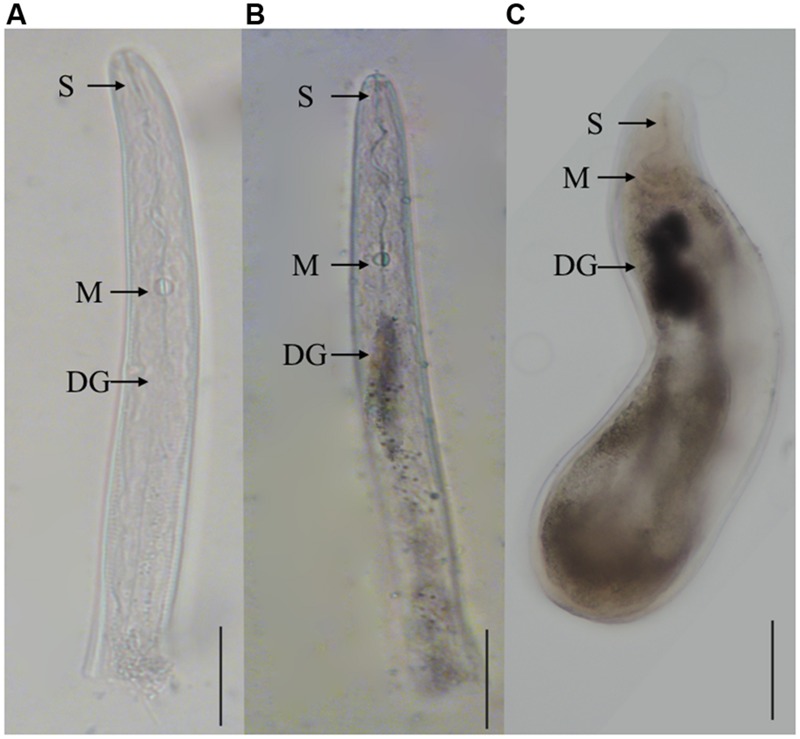
***In situ* hybridization of the *Misp12* transcripts in parasitic second-stage juveniles and females. (A)** The sense *Misp12* DIG-labeled cDNA probes as a negative control in parasitic second-stage juveniles. **(B,C)** Signal of antisense probes localized within the dorsal esophageal gland (DG) in parasitic second-stage juveniles and females. The DG, metacorpus (M), and stylet (S) are indicated with arrows. Scale bar = 50 μm.

*In situ* mRNA hybridization was used to determine the tissue localization of *Misp12* in the nematode. No hybridization signals were detected in the control treatment when using the DIG-labeled sense cDNA probe (**Figure 3A**). And the antisense *Misp12* cDNA probe hybridized with mRNA within the dorsal esophageal gland cell of parasitic J2 and female (**Figures 3B,C**). The results indicated that *Misp12* was synthesized at dorsal esophageal gland.

### Misp12 was Secreted into the Cytoplasm of Plant Cell

**FIGURE 4:**
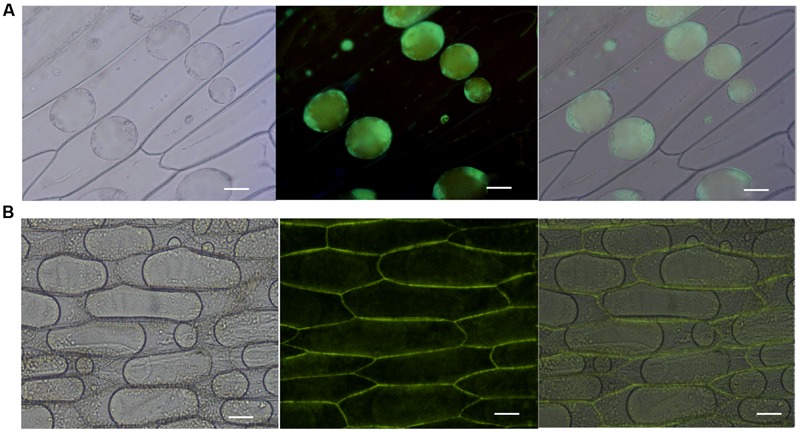
**Subcellular localization of Misp12 in the plant cell. (A)** *Agrobacterium tumefaciens* cells carrying 35S:ΔspMisp12:GUS:GFP fusion were transiently expressed in onion epidermal cells. The images present sequentially: bright-field, dark-field, and composite image. **(B)** *Agrobacterium tumefaciens* cells carrying 35S:Misp12:GUS:GFP fusion. The images present sequentially: bright-field, dark-field, and composite image. Scale bar = 100 μm.

To evaluate the subcellular localization of Misp12 *in planta, Misp12* with or without signal peptide gene fragment fused with e*GFP* and *GUS* genes was transiently expressed in onion epidermal cells. The result showed that Misp12 without signal peptide was located in the cytoplasm (**Figure 4A**). However, full-length Misp12 was observed in the apoplast (**Figure 4B**). Free eGFP was present in the cytoplasm of the cell (**Supplementary Figure S2**). The results showed that Misp12 was located at the cytoplasm of the plant cell during infection.

### *In Planta* RNAi of *Misp12* Attenuates Nematode Parasitism

**FIGURE 5:**
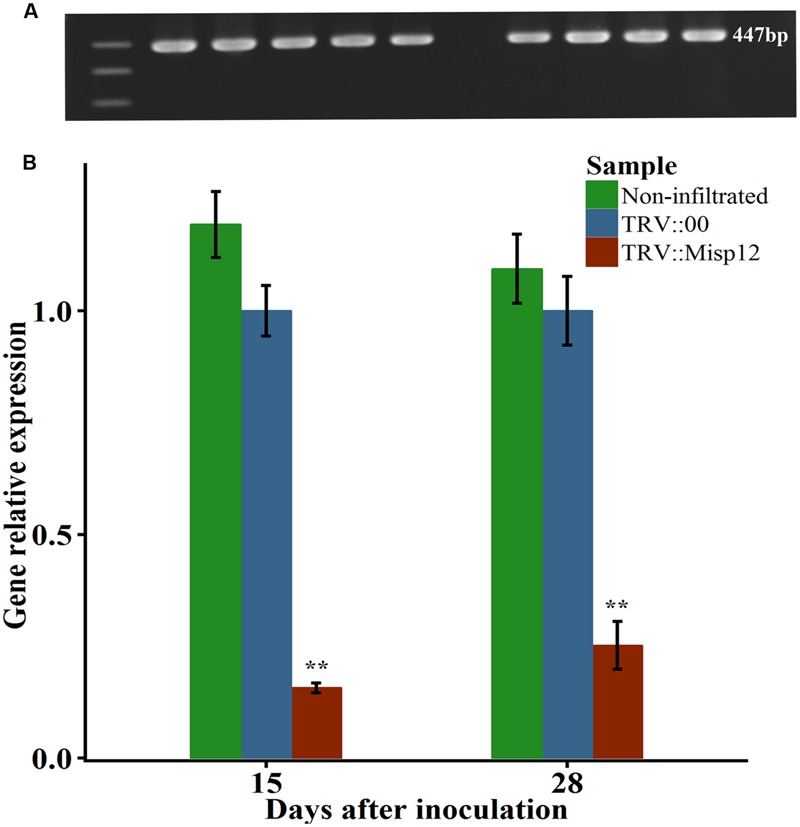
**The *Misp12* silence efficiency. (A)** The figure of TRV coat gene sequence by agarose gel electrophoresis. **(B)** qRT-PCR assays for relative expression levels of *Misp12* in *M. incognita* collected from non-infiltrated plants, TRV-vector transformed control roots (TRV::00) and *Misp12*-silencing root (TRV::Misp12); *Actin* and *18S* was amplified as control. qRT-PCR experiments were repeated thrice with the similar results. Each bar value represents the mean ± SD of *n* > 10. Columns for the same time point or treatment marked with ^∗∗^ are significantly different (*P* < 0.01) from each other based on Student’s *t*-test.

A virus-induced gene silencing (VIGS) technique was utilized to silence *Misp12* in nematodes and the consequential effects on parasitism were evaluated. TRV-based expression vectors were employed to drive the expression of dsRNA complementary to *Misp12* in *N. benthamiana*. A unique 249 bp fragment amplified from *Misp12* cDNA named *Umisp12* was ligated into vector pTRV_2_ to generate the expression constructs TRV_2_::*Misp12*. The leaves of *N. benthamiana* were co-infiltrated with cultures of recombinant *Agrobacterium* strains containing expression vectors of pTRV_1_ with TRV_2_::*Misp12* or with empty vector pTRV_2_::00. At 5 DAI, the 447 bp TRV coat gene was detected by qRT-PCR in plant roots (**Figure 5A**). qRT-PCR analysis demonstrated that the TRV::Misp12-infiltrated plants showed a 75–80% reduction in *Misp12* transcript level at 15 and 28 DAI when compared to the empty vector-infiltrated plants or non-infiltrated plants (**Figure 5B**).

**FIGURE 6:**
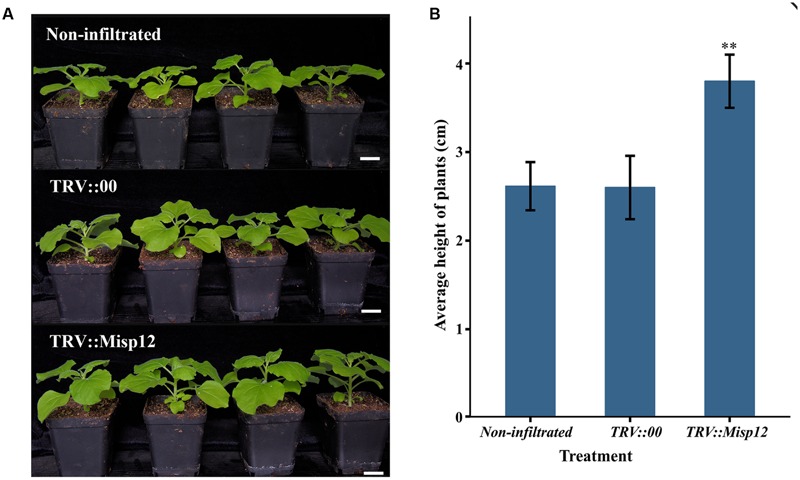
**The plants comparison of *Misp12*-silencing plants and the control plant. (A)** Photograph of non-infiltrated plants, TRV-vector transformed control plants (TRV::00) and *Misp12*-silencing plants (TRV::Misp12) at 20 DAI. Scale bar = 2 cm. **(B)** The average height of non-infiltrated plants, TRV::00 control plants and *Misp12*-silencing plants at 20 DAI. Columns for the treatment marked with the “∗∗” are significantly different (*P* < 0.01) from others based on Student’s *t*-test.

**FIGURE 7:**
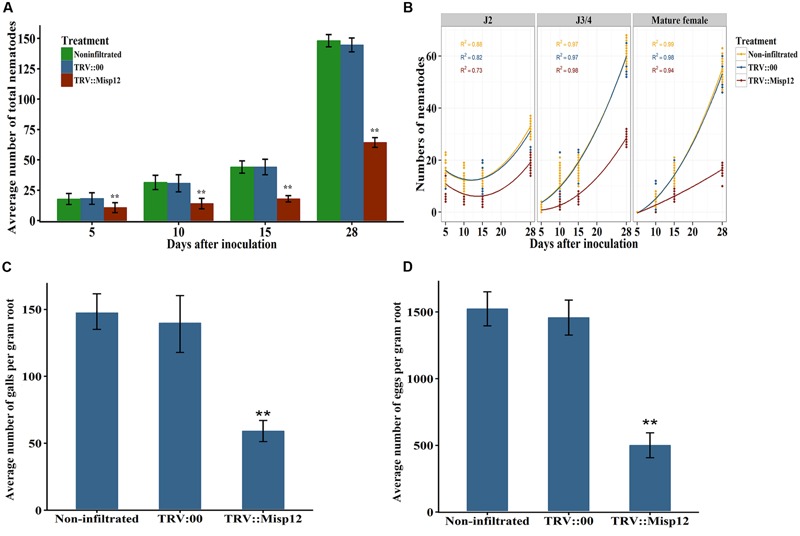
**Effect of *in planta* RNAi of *Misp12* on *M. incognita*. (A)** Numbers of total nematodes in the *N. benthamiana* roots at various DAI, as indicated. **(B)** Numbers of nematodes at different stages in the *N. benthamiana* roots at various DAI. **(C,D)**, Numbers of galls and eggs, respectively, in the *N. benthamiana* roots at 45 DAI. Each column represents the mean of three independent experiments with standard deviations. Columns for the same time point or treatment marked with ^∗∗^ are significantly different (*P* < 0.01) from each other based on Student’s *t*-test.

Comparing the parasitism of nematodes in transgenic *N. benthamiana* and non-transgenic plants, we found that the *Misp12*-RNAi plants grow better than the non-transgenic plants (**Figure 6**), and the RNAi of *Misp12* could significantly affected the galls formation, the numbers of eggs and nematodes (**Figure 7**).

In TRV::*Misp12-*infiltrated plants roots, the number of parasitic nematodes exhibited much fewer mature females than non-infiltrated plants or plants infiltrated with TRV::00. At 5, 10, 15, and 28 DAI, the numbers of nematodes in the *Misp12*-RNAi transgenic lines reduced in a range from 41 to 59% when compared to the empty vector-transformed lines (**Figure 7A**). Moreover, the distribution of nematodes in different developmental stages in the root trended differently between the *Misp12*-RNAi and the control plants. For migratory parasitic J2s, the trend of nematode numbers in the *Misp12*-RNAi plants was similar to the control plants. While the J3/J4 and females in the *Misp12-*RNAi plants were sharply decreased at 28 DAI when compared to the control plants. Especially, the number of the mature females decreased by 69% in the *Misp12*-RNAi plants (**Figure 7B**). Additionally, at 45 DAI, both the size and amount of root galls in the *Misp12-*RNAi plants showed a significant reduction when compared to the empty vector-transformed lines and non-transgenic plants (**Figure 7C**). Likewise, the number of eggs showed a similar situation with 63% reduction rate (**Figure 7D**). These results suggested that *Misp12* could promote the abilities of the nematode, especially at latter parasitism.

### Higher Expression Level of Defense Genes in the *Misp12*-RNAi Transgenic Plant Roots

**FIGURE 8:**
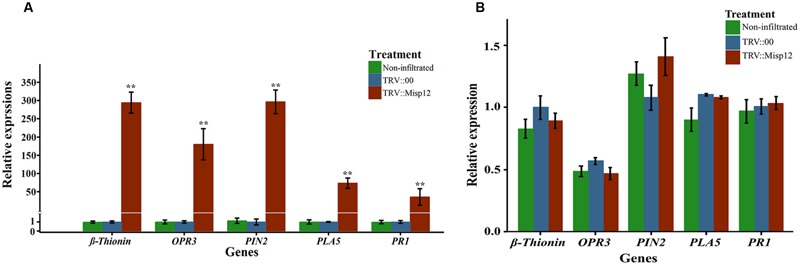
**Relative expression levels of genes involved in JA and SA signaling defensive pathways. (A)** Expression level of five JA and SA signal pathway mark genes in *Misp12*-silencing root at 28 DAI with *M. incognita*. **(B)** Expression level of the same mark genes in *Misp12*-silencing root at 5 DAI. The graph showed the mean and standard error of the relative amount of transcripts of these genes in *Misp12*-silencing root (TRV::Misp12) in comparison with TRV-vector transformed control roots (TRV::00) growing under the same conditions. Each reaction was performed in triplicate and the results represented the mean of three independent biological replicates. Columns for the same time point or treatment marked with ^∗∗^ are significantly different (*P* < 0.01) from each other based on Student’s *t*-test.

At 5 and 28 days after nematodes infection, the expression level of JA and SA signaling pathway defense genes were evaluated in the *Misp12*-RNAi transgenic plant roots and the control plant roots. At 28 DAI, three genes of *OPR3, Pin2* and β*-thionin* genes in JA signaling pathway has 190-, 280-, and 280-fold up-regulation, respectively, when compared to the empty-vector transgenic plant or non-infiltrated plant roots. Consistently, the expression levels of SA marker genes *PAL5* and *PR1* were increased by 62- and 37-fold, respectively, at 28 DAI (**Figure 8A**). At 5 DAI, the JA and SA signaling pathway defense genes had no significant change compared with empty-vector transgenic plant or non-infiltrated plant roots (**Figure 8B**). These results indicated that Misp12 could suppress the plant defense response to nematodes at the latter stages of nematode infection.

### Down-Regulation of Defensive Genes in *Misp12-*exprssing Plant

**FIGURE 9:**
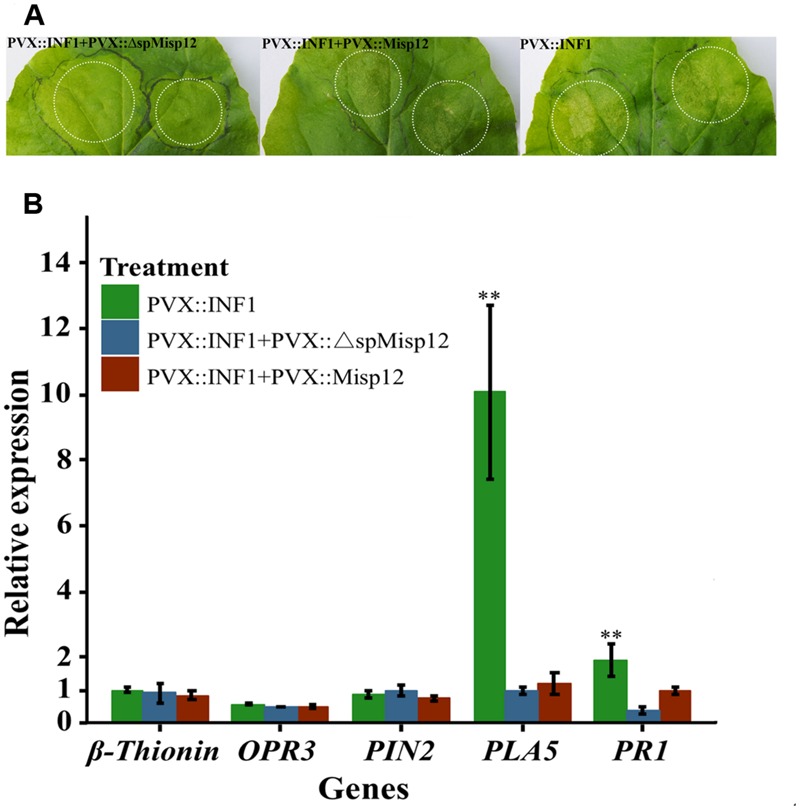
**Misp12 suppresses *INF1*-triggered cell death **(A)** Graph showing the necrosis triggered by co-infiltration.** *N. benthamiana* leaves were infiltrated *Agrobacterium* cells carrying the *Misp12* gene with or without signal peptide 24 h before infiltration with *Agrobacterium* cells carrying the *INF1* gene (PVX::Misp12+PVX::INF1 or PVX:: ΔspMisp12+PVX::INF1), the *INF1* genes (PVX::INF1) were infiltrated alone as control. Representative images were acquired 2 days after the last infiltration. **(B)** Expression level of five JA and SA signal pathway mark genes mark genes in *Misp12*-expressing plant at 2 DAI with PVX::INF1. The plants growing under the same conditions. Each reaction was performed in triplicate and the results represented the mean of three independent biological replicates. Columns for the same time point or treatment marked with ^∗∗^ are significantly different (*P* < 0.01) from each other based on Student’s *t*-test.

At 2 days after PVX::INF1 infiltrated into the leaves of *N. benthamiana*, the Misp12 without signal peptide could obviously suppress programmed cell death triggered by INF1 (PT-PCD), and the Misp12 with signal peptide may partially suppressed PT-PCD. (**Figure 9A** and **Supplementary Figure S3**). Additionally, the expression levels of SA marker genes *PAL5* and *PR1* were strongly induced in control plants and were repressed in plants overproducing Misp12 (**Figure 9B**). However, the JA-related defense genes *OPR3, Pin2 and β-thionin* had no significant changes (**Figure 9B**).

## Discussion

Since the completion of genome sequencing of different kinds of nematodes, tremendous progress has been made toward the building of effectors repertoire. Current studies mainly focused on the early parasitism of plant-nematode interactions. Most of the identified effectors were highly expressed in the migratory parasitic J2s, which suggested that these effectors are involved in the root invasion, larvae migration and the formation of the GCs (Rehman et al., 2016). The GCs need to be maintained throughout of the whole life of the nematodes, so some effectors must exist at the later parasitism. Here, we reported a novel potential effector, *Misp12*, that might play a key role at the later parasitic stage by using VIGS and transcription analysis.

It is reported that an abundance existence of unique genes contributes to *M. incognita’s* wide host range, a range that exceeds other parasitic nematodes (Bird et al., 2009). The BLAST results indicated that no other similar sequences of *Misp12* exists in the other organisms, and the further gene amplification of *M. incognita* collected from eight different areas of China showed that *Misp12* had only a few nucleotide mutations. These results suggested that *Misp12* was highly conserved and specific in *M. incognita* and might be involved in the improvement of host range.

In parasitic nematodes, mature effector proteins need the signal peptide to be imported into the endoplasmic reticulum of the cells and then to be secreted into the host via the nematode stylet (Elling et al., 2007; Sacco et al., 2009). Based on the bioinformatics tools analysis, the Misp12 protein was predicted to be a potential effector. Consistent with this prediction, the subcellular localization of Misp12 *in planta* indicated that the Misp12 protein was delivered to the cell cytoplasm and targeted cell cytoplasmic proteins. Additionally, *in situ* mRNA hybridization suggested that the *Misp12* was specifically expressed in the dorsal esophageal gland, which confirmed the results of a previously report (Huang et al., 2003). Esophageal is the common origin of nematode secretory proteins localization (Davis et al., 2004). Thus, consistent with previously report (Huang et al., 2003), we deduced that Misp12 was a secretory protein and a novel effector of *M. incognita*. It is believed that the subventral glands show activity at the stage of nematode penetration and migration, whereas the dorsal glands tend to respond during the formation and maintenance of the nematode feeding cells in the sedentary nematode life stages (Davis et al., 2008). The expression level of *Misp12* was significantly greater at the mature female stages. Hence, as a secretory protein derived from the dorsal esophageal gland, *Misp12* might be involved in the maintenance of GCs during the latter parasitism.

VIGS is an alternate useful tool to study target genes as the knock-out of specific nematode genes is not feasible for plant-parasitic nematodes at present (Becker and Lange, 2010; Senthil-Kumar and Mysore, 2011; Lee et al., 2012). To observe the consequential effects of *Misp12* on nematode parasitism, VIGS was employed to silence the *Misp12* gene. It resulted in fewer galls, eggs, and parasitic nematodes in the gene silenced-plant roots than that in the negative control plants group. Notably, gene silencing induced numbers of J3/J4 and mature female nematodes a more significant reduction. The numbers of J3/J4 showed a 65% reduction and the mature female showed a 69% reduction at 28 DAI. These results were consistent with the dynamics of *Misp12* expression levels. Thereby, we concluded that *Misp12* played a critical role during latter parasitism, especially at the mature stage of *M. incognita.*

Root-knot nematodes are biotrophic pathogens and the success of infection depends on the ability of the pathogen to overcome the plant defense system. SA and JA hormones can activate the plant defense system (Grant and Jones, 2009; Pieterse et al., 2009; Yan and Dong, 2014; Yang et al., 2015; Zhang et al., 2015b) and play crucial roles in plant defense responses against RKNs and other parasitic nematodes (Molinari and Loffredo, 2006; Bhattarai et al., 2008; Molinari et al., 2014). Several effectors that influence the plant defense responses have been characterized (Quentin et al., 2013; Rehman et al., 2016). Chorismate mutases, which are secreted by the RKNs, can affect the plant shikimate pathway, thereby decreasing the synthesis of SA and phytoalexin through competition with chorismate, and can prevent the triggering of host defense (Huang et al., 2005b; Long et al., 2006). The effectors Hs10A06 from *Heterodera schachtii* targets A*rabidopsis* spermidine synthase and suppresses the SA signaling pathway in the syncytia (Hewezi et al., 2010). And Mi-CRT from *M. incognita* also acts on the SA signaling pathway to promote the nematodes parasitism (Jaouannet et al., 2013). In this study, the results showed that *PR1* and *PAL5* were up-regulated in the virus-induced silencing of *Misp12* plants and down-regulated in the *Misp12*-expressing plants. It is reported that *PR-1* was highly induced in *Arabidopsis* roots after *M. incognita* infection (Hamamouch et al., 2011). These may imply that SA signaling pathways could be manipulated by Misp12 in the root cells to support nematode parasitism at the latter stages.

Jasmonic acid pathways also play a major role in the defense against the RKNs (Nahar et al., 2011). The effector Mi-CRT mentioned above can also suppressed the defense genes from the JA pathway (Jaouannet et al., 2013). Mj-FAR-1, a secreted fatty acid and retinol binding proteins from *M. javanica*, could manipulate the lipid based signaling to suppress the JA related defense gene (Iberkleid et al., 2013). In this study, the *proteinase inhibitors*(*Pin2*), *12-oxophyto-dienoate reductase* (*OPR3*) and β*-thionin* genes, associating with the JA metabolic pathway (Wasternack, 2007; Fujimoto et al., 2011) were significantly up-regulated in the virus-induced silencing of *Misp12* plants, indicating the *Misp12* gene could manipulate these defense genes. Interestingly, it was reported that β*-thionin* is involved in inhibiting mammalian cell growth by membrane permeabilization (Li et al., 2002) and able to inhibit insect amylases and proteinases activity (Li et al., 2002; Melo et al., 2002). Moreover, previous researches has shown that proteinase inhibitors (*Pin2*) respond to wounding when attacked by tobacco hornworm larvae (Howe et al., 1996), and predicated that protease inhibitor could enhance plant resistance to nematodes (McPherson and Harrison, 2001). Thus, up-regulation of β*-thionin* and *Pin2* found in the virus-induced silencing of *Misp12* plant roots might also contribute to the suppression of nematode parasitism. However, in the *Misp12*-expressing plants, in which the *INF1* gene was inoculated, the JA-related defense genes had no significant changes; this may be due to JA defense genes not being active in the progression of PT-PCD.

Interestingly, Misp12 with and without signal peptide have different localizations in the plant cell, but both of them suppressed programed cell death (PCD) triggered by *INF1* through transient expression in *N. benthamiana*. Recently, two secreted nematode effectors, *MeTCTP* from *M. enterolobii* and *MiMsp40* from *M. incognita*, were demonstrated to suppress the PCD triggered by BAX (Niu et al., 2016; Zhuo et al., 2016), but both effectors were up-regulated during the early parasitic stages. The *Misp12* up-regulated at the J3/4 and female stages, which suggested that the *Misp12* might suppress the PCD at the nematode mature stages to maintain the GCs for nutrients.

Taken together, although the mechanism of host defense suppression by Misp12 and the plant target for Misp12 remains unclear, our data indicated that Misp12 protein has a potential through down-regulation of SA and JA-dependent defense responses genes to promote the latter parasitism of *M. incognita* during the mature stages. This study revealed a novel effector *Misp12*, which functions at the latter nematode parasitic stage, and it would be a new complement to the effectors of the parasitic nematode. This gene plays a key role in manipulating plant defense signal responsive genes to maintain the GCs, thus promoting successful parasitism in the host plant. Furthermore, *Misp12* could be a new target gene to control nematode disease.

## Author Contributions

YX and XX initiated and designed the research. JX and SL wrote the manuscript. JX, SL, and CM performed experiments and analyzed data. YX, XX, and GW reviewed the paper. All authors read and approved the final manuscript.

## Conflict of Interest Statement

The authors declare that the research was conducted in the absence of any commercial or financial relationships that could be construed as a potential conflict of interest.

## Footnotes

1 http://www6.inra.fr/meloidogyne_incognita

## Supplementary Material

Figure S1 **The alignment of Misp12 from 8 different area of China**.

Figure S2 **The free eGFP location in the onion cells**.

Figure S3 ***Misp12* suppresses *INF1*-triggered cell death using *A. tumefaciens* cells with OD_600_ = 1.0 at 2 DAI**.
