# Supplementary material for: A Novel Meloidogyne incognita Effector Misp12 Suppresses Plant Defense Response at Latter Stages of Nematode Parasitism
Source: Front Plant Sci. 2016 Jun 30;7:964. doi: 10.3389/fpls.2016.00964 (PMC4927581; doi:10.3389/fpls.2016.00964)
Supplement: Supplementary file 1 [file Table_1.DOCX]

**Table S1. List of primers used in this study.**

| **Primer name** | **Primer sequence (5'→3')** |
| --- | --- |
| RACE12S1 | CGGATACCAAGGAAAATAAC |
| RACE12S2 | CCTGTCACTAACATACCATC |
| Misp12QS | CATCAAACAATCTCCTCAAC |
| Misp12QA | GGAGACAGATATTGTGAAGC |
| qPCRmi12S | CCTGTCACTAACATACCATCCG |
| qPCRmi12A | GAGGAGGGCAAACATTAGG |
| QactinS | GTGATGACTTGACCGTCAGGC |
| QactinA | TGACTCTGGAGATGGTGTTACG |
| 18SF | CGAGGAGGTAGTGACGAG |
| 18SR | CGAAGGGTTCCAAGGTAT |
| Misp12HS | TCAAATGGAACCTCAACAAT |
| Misp12HA | CGGATACCAAGGAAAATAAC |
| 12S | *C*ATGTCCATCTTCCTTACTTCTGC |
| 12BamHI-A | *GCGGATCC*TTACTTCCGGGGCAACATGA |
| 12nospS | *C*GAGGGTGCAGGCGATC |
| 12nospBamHI-A | *ATGGATCC*TTACTTCCGGGGCAACAT |
| GUSBamHIS | *GCGGATCC*ATGTTACGTCCTGTAGAAACCC |
| GUSA | TCATTGTTTGCCTCCCTGCTGCGG |
| GUSHindIIIA | *AAGCTT*TCATTGTTTGCCTCCCTGCTGCGG |
| 12EcoRI-S | *GCGAATTC*ATGTCCATCTTCCTTACTTCTGC |
| 12nospEcoRI-S | *ATGAATTC*GAGGGTGCAGGCGATC |
| U12RNAiS | *TCTAGA*CCTGTCACTAACATACCATC |
| U12RNAiA | *AGTACT*AATTAGAGACTGCTCCTGTT |
| TRVcpF | CTGGGTTACTAGCGGCACTGAATA |
| TRVcpR | TCCACCAAACTTAATCCCGAATAC |
| NbactinS | TCCTGATGGGCAAGTGATTAC |
| NbactinA | TTGTATGTGGTCTCGTGGATTC |
| NbGAPDHS | AGCTCAAGGGAATTCTCGATG |
| NbGAPDHA | AACCTTAACCATGTCATCTCCC |
| PR1S | GGTCAATACGGCGAAAACCT |
| PR1A | ACCCTAGCACATCCAACACG |
| PAL5S | ACGTCTTTGCTTACGCTGAT |
| PAL5A | CGTGATTCTTGCACTCTCGA |
| PIN2S | GGAAAGGTTGCAAACTTGTCCGTA |
| PIN2A | AAGCTACAAGGCCACTATTTAGCA |
| OPR3S | TATATCCGCCACTACAAATA |
| OPR3A | TAAACAAACTCCAACTCCAT |
| *r-thionin*S | GAGATGGGAACAATGGTG |
| *r-thionin*A | GATATTCGGGGAAAGACA |
