# Supplementary figures and images for: A Novel Meloidogyne incognita Effector Misp12 Suppresses Plant Defense Response at Latter Stages of Nematode Parasitism
Source: Front Plant Sci. 2016 Jun 30;7:964. doi: 10.3389/fpls.2016.00964 (PMC4927581; doi:10.3389/fpls.2016.00964)

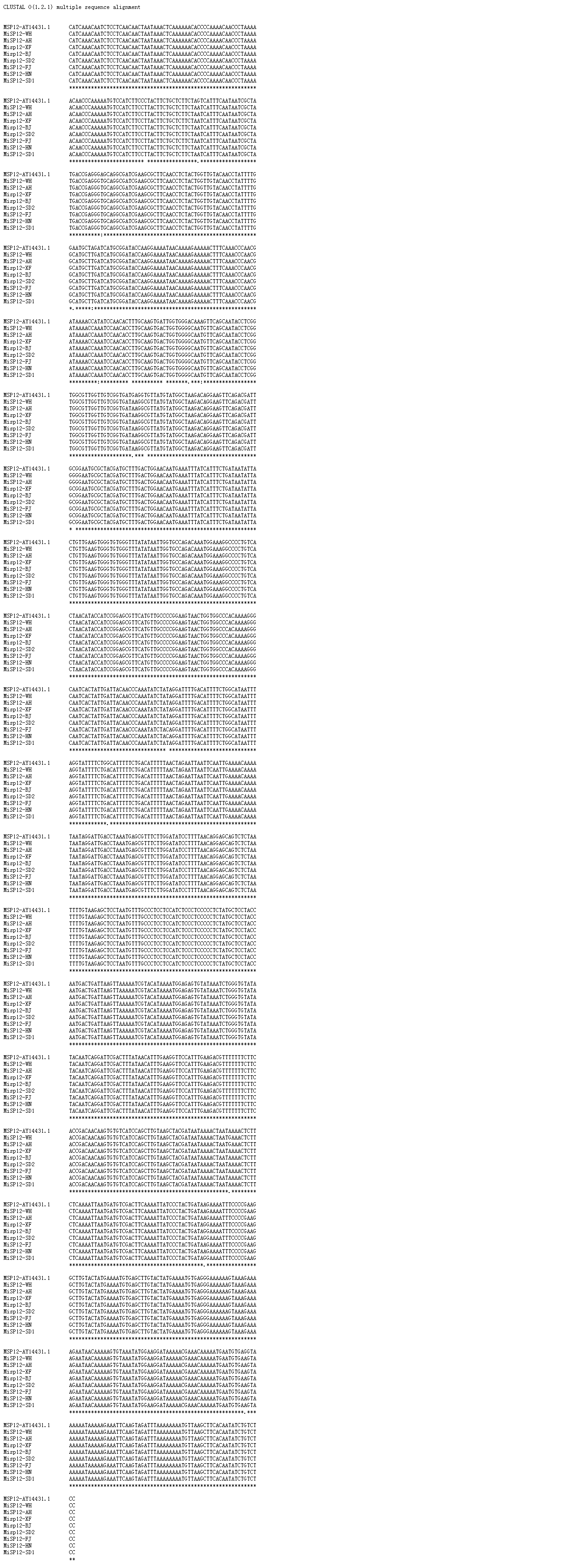

Supplement: Figure S1 — The alignment of Misp12 from 8 different area of China. [file Image_1.PNG]

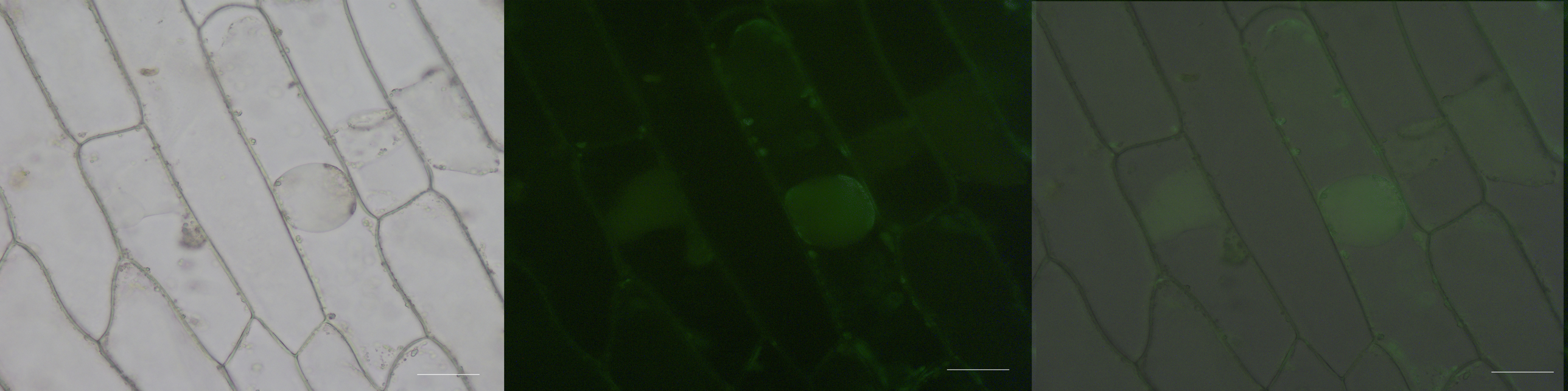

Supplement: Figure S2 — The free eGFP location in the onion cells. [file Image_2.JPEG]

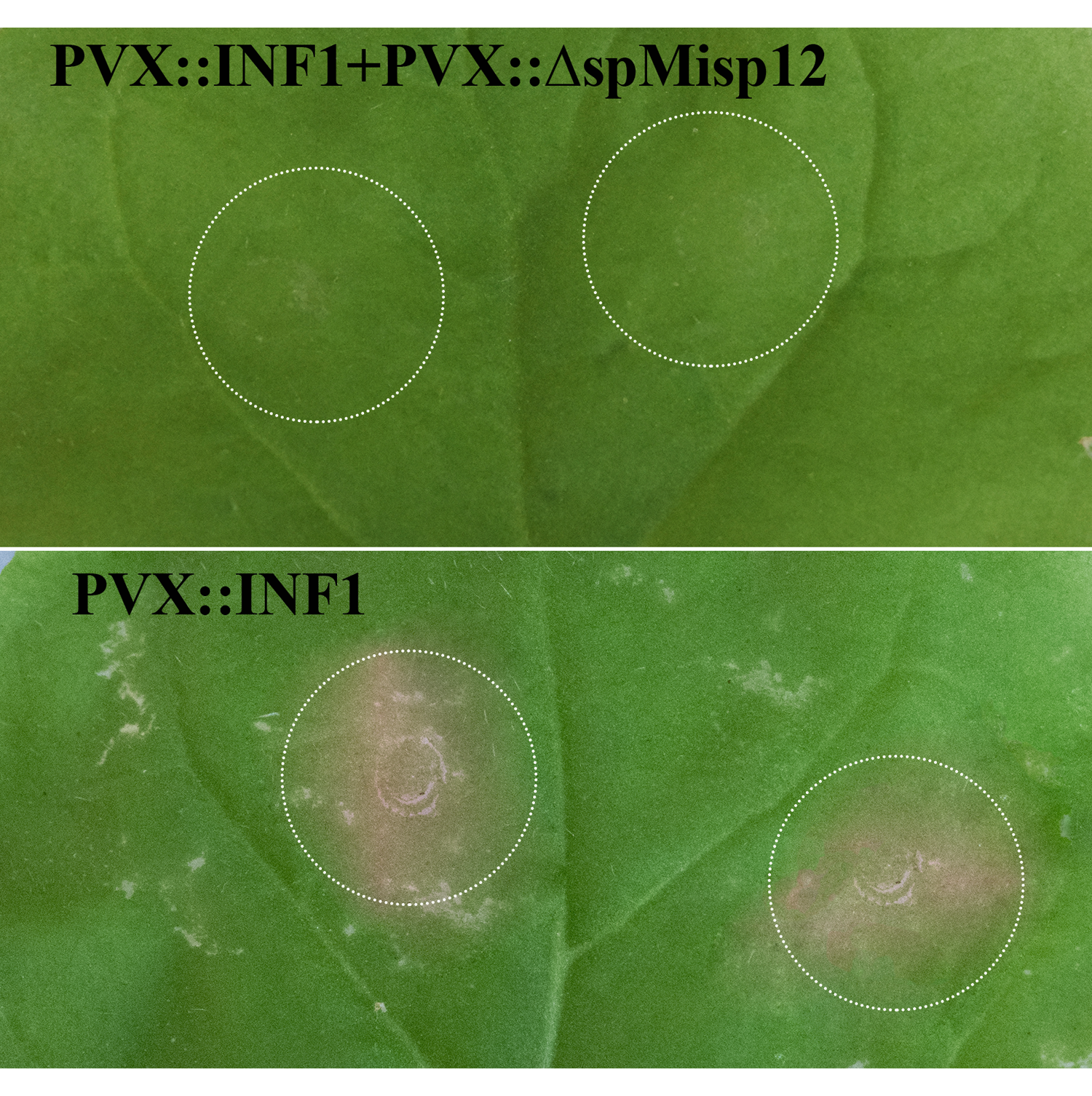

Supplement: Figure S3 — Misp12 suppresses INF1-triggered cell death using A. tumefaciens cells with OD600 = 1.0 at 2 DAI. [file Image_3.TIF]
